# NiO–Ba_0.95_Ca_0.05_Ce_0.9_Y_0.1_O_3−__δ_ as a Modified Anode Material Fabricated by the Tape Casting Method

**DOI:** 10.3390/ma15072489

**Published:** 2022-03-28

**Authors:** Magdalena Dudek, Bartłomiej Lis, Ryszard Kluczowski, Mariusz Krauz, Magdalena Ziąbka, Marcin Gajek, Alicja Rapacz-Kmita, Michał Mosiałek, Piotr Dudek, Dorota Majda, Andrzej Raźniak

**Affiliations:** 1 Faculty of Energy and Fuels, AGH University of Science and Technology, Av. A. Mickiewicza 30, 30-059 Krakow, Poland; blis@agh.edu.pl (B.L.); razniak@agh.edu.pl (A.R.); 2 Institute of Power Engineering–Research Institute, Mory 8, 01-330 Warsaw, Poland; kluczowski@cerel.pl (R.K.); krauz@cerel.pl (M.K.); 3 Faculty of Materials Science and Ceramics, AGH University of Science and Technology, Av. A. Mickiewicza 30, 30-059 Krakow, Poland; ziabka@agh.edu.pl (M.Z.); mgajek@agh.edu.pl (M.G.); kmita@agh.edu.pl (A.R.-K.); 4 Jerzy Haber Institute of Catalysis and Surface Chemistry PAS, Niezapominajek 8, 30-239 Krakow, Poland; nbmosial@cyfronet.pl; 5 Faculty of Mechanical Engineering and Robotics, AGH University of Science and Technology, Av. A. Mickiewicza 30, 30-059 Krakow, Poland; pdudek@agh.edu.pl; 6 Faculty of Chemistry, Jagiellonian University, Gronostajowa 2, 30-387 Krakow, Poland; majda@uj.edu.pl

**Keywords:** high-temperature protonic ceramic fuel cells, anode, cermet, tape casting, BCY

## Abstract

The development of new chemically resistant anodes for protonic ceramic fuel cells (PCFCs) is urgently required to avoid the costly deep hydrogen purification method. Ba_0.95_Ca_0.05_Ce_0.9_Y_0.1_O_3−__δ_ (5CBCY), which is more chemically resistant than BaCaCe_0.9_Y_0.1_O_3__−__δ_, was here tested as a component of a composite NiO–5CBCY anode material. A preparation slurry comprising 5CBCY_,_ NiO, graphite, and an organic medium was tape cast, sintered and subjected to thermal treatment in 10 vol.% H_2_ in Ar at 700 °C. Differential thermal analysis, thermogravimetry, quadrupole mass spectrometry, X-ray diffraction analysis, scanning electron microscopy, the AC four-probe method and electrochemical impedance spectroscopy were used for the investigation. The electrical conductivity of the Ni–5CBCY in H_2_–Ar at 700 °C was 1.1 S/cm. In the same gas atmosphere but with an additional 5 vol.% CO_2_, it was slightly lower, at 0.8 S/cm. The Ni–5CBCY cermet exhibited repeatable electrical conductivity values during Ni-to-NiO oxidation cycles and NiO-to-Ni reduction in the 5CBCY matrix, making it sufficient for preliminary testing in PCFCs.

## 1. Introduction

Protonic ceramic fuel cells (PCFCs) have been investigated as an option to lower the operating temperature of solid oxide fuel cells (SOFCs) in the 400–800 °C range.

Perovskite materials based on BaCe_0.9_Y_0.1_O_3−__δ_ (BCY) are considered promising proton-conducting electrolytes for application in PCFCs [1,2]. However, BCY-based proton-conducting electrolytes have the major drawback of limited chemical stability in hydrogen gas atmospheres containing CO_2_ or H_2_O impurities [3]. The conversion of fossil fuels, biomass or organic waste materials produces a hydrogen-rich gas mixture.

Therefore, a hydrogen purification process and the removal of impurities (such as CO_2_, CO, SO_x_, and H_2_O) are required. Hydrogen is a clean fuel source, but its purification remains a challenge. The development of new chemically resistant components (electrolytes and anodes) is urgently required to avoid the costly deep hydrogen purification method, especially for fuel cell applications [4,5].

A second approach is to modify the physicochemical properties of well-known BCY-based materials to improve their chemical resistance in gas atmospheres containing H_2_O [6,7]. Previously, our research has shown that the co-doped electrolytes CaO–Y_2_O_3_–BaCeO_3_ and SrO–Y_2_O_3_–BaCeO_3_ (chemical formula: Ba_1−x_M_x_Ce_0.9_Y_0.1_O_3−δ_, M = Ca, Sr and 0 < x < 0.05) exhibited improved chemical stability in H_2_O–H_2_–Ar or CO_2_–H_2_–Ar gas atmospheres compared with the original composition of BaCe_0.9_Y_0.1_O_3_. The investigated co-doped materials exhibited the same level of electrical conductivity as BCY in H_2_–Ar gas atmospheres, but their chemical resistance to CO_2_ attacks improved compared with the BCY samples [8,9]. The co-doped gas-tight Ba_0.95_Ca_0.05_Ce_0.9_Y_0.1_O_3−__δ_ (5CBCY) samples were prepared using gel and tape casting (100–500 μm) and tested in electrolyte-supported SOFCs, with positive results [10,11].

Anode-supported SOFCs (A-SOFCs) have a well-known ceramic fuel cell structure in which the anode acts as structural support for the fuel cells. This offers the possibility of applying a thin electrolyte layer, thus reducing each single fuel cell’s internal ohmic resistance. In the classical construction of A-SOFCs, the anode support layer is the thickest layer (approximately 0.5–1 mm) among the layered structure of the fuel cell.

The primary function of the anode in ceramic fuel cells is to promote the electrochemical oxidation of the fuel. If hydrocarbons (methane or others) are used as fuel, the anode may have the additional function of internally reforming or oxidating the fuel. In the case of PCFCs involving ceramic proton conductors, developing and constructing an anode support is also desirable due to the possibility of obtaining higher power and current density from single fuel cells compared with that obtained in the construction of electrolyte-supported PCFCs [12,13].

Nickel-based cermets, in which Ni particles are distributed in an electrolyte matrix, are the most commonly used anodes in SOFCs and PCFCs. Nickel is a cost-effective catalyst for the electrochemical oxidation of hydrogen, and it also leads to high electronic conductivity, which is crucial for electrode applications in SOFCs and PCFCs.

In cermet anodes, Ni is usually randomly distributed in a matrix of oxide-conducting solid electrolyte (for SOFCs) or ceramic proton-conducting electrolyte (for PCFCs). The commercial application of Ni-based cermets is well known for SOFCs and is in the development stage for PCFCs [14,15]. Essoumhi et al. [16] investigated symmetrical cells with Ni–BCY/BCY/Ni–BCY cermet anodes. A thin layer of BCY electrolyte (approximately 20 μm) was applied, and a total specific resistance of 0.4 Ω cm^2^ at 600 °C was achieved for optimised symmetrical cells. Chevallier et al. [17] devised a new technique for preparing NiO–BCY powders obtained in one step using wet chemical methods as an alternative to the mechanical mixing of NiO and BCY powders.

Some data in the literature also pertain to the laboratory-scale fabrication of A-SOFCs. Zuic et al. [18] investigated proton-conducting BCY thick films and deposited them on cermet anodes made of Ni–yttrium-doped barium cerate using the electrophoretic deposition technique. The BCY powders were prepared using the citrate–nitrate auto-combustion method, while the cermet anodes were formed using an evaporation and decomposition solution and suspension method. In another study, a prototype fuel cell was prepared by depositing a composite La_0.8_Sr_0.2_Co_0.8_Fe_0.2_O_3_ (LSCF)–BaCe_0.9_Yb_0.1_O_3−δ_ (YbBC) cathode on a co-sintered half-cell. Fuel cell tests performed at 650 °C on the single-cell prototypes revealed a maximum power density of 174 mW cm^−2^ [19,20]. Several possible methods have been developed for fabricating Ni–BCY or Ni–BZY anode materials, including dry pressing, dip coating, tape casting, electrodeposition, gel casting and high-pressure injection moulding [21,22,23]. However, to date, no study has focussed on the preparation of a modified anode involving co-doped barium–yttrium cerates and metallic Ni with parallel investigations into their physicochemical properties aimed at improving the performance of these materials in a hydrogen gas atmosphere containing 1–5 vol.% CO_2_ as an impurity.

The goal of this paper is to present the results of our research on fabricating NiO–5CBCY porous samples using the tape casting method in a non-aqueous environment. Focus is given to the unique quality of flat-parallel anode supports and the determination of variations in phase composition, microstructure, grain size and pore distribution for sintered NiO–5CBCY samples and anode-supporting Ni–5CBCY samples. The electrical and electrochemical properties of the Ni–5CBCY obtained in this process are also discussed.

## 2. Experiments

### 2.1. Preparation of Ni–5CBCY Cermet Using the Tape Casting Method

The starting components for preparing the Ni–5CBCY included commercial NiO and 5CBCY powder synthesised via a solid-state reaction. The physicochemical properties of the 5CBCY powder have been previously described [24,25]. The main parameters of the commercial NiO powder (Novamet, Lebanon, TN, USA) included an average particle size of d_50_ = 1–2 μm, an apparent density of 1.1 g/cm^3^ and a specific surface area S_w_ of 3–4 m^2^/g. Graphite (Aldrich, Germany) was used as a pore former to obtain adequate porosity during the fabrication of the cermet electrode. The applied procedure was based on an attempt to develop technological guidelines for producing Ni–5CBCY anode supports using the slurry casting method. The organic medium consisted of polyvinyl butyral (PVB), which acts as a binder, and W5000 (Zschimmer & Schwarz, Germany), which acts as a plasticiser. A mixture of methyl ethyl ketone and ethanol was used as a solvent. Table 1 outlines the masses of the main components used to fabricate the NiO–5CBCY tape sheets.

The mixture of inorganic components comprised 38 wt% 5CBCY, 57 wt% NiO and 5 wt% graphite (as a pore former), which were ground in a polyethene ball mill with an organic medium at a powder:organics mass ratio of 54:46.

The preparation of the NiO–B5CBCY porous anode foils consisted of the following steps listed as I to V. The duration of each step is given in parentheses, and the estimated total time for stages I–V was over 120 h:

- Pre-mix all the reagents listed in Table 1 and prepare the suspension components (~0.5 h).
- Intensively homogenise all of the reagents by grinding in a ball mill placed on high-speed rollers (~48 h).
- Further homogenise the prepared slurry from stage II at a reduced rotation speed, degassing the slurry (~48 h).
- Transfer the slurries to the laboratory tape caster device and begin the tape casting process for NiO–5CBCY. The gap between the cast squeegee and tape-casting table surface was 2 mm. The green tape was cast at a rate of 2 cm/s on a polytetrafluoroethylene (PTFE) surface (~0.5 h).
- Dry the casted NiO–5CBCY tape in an air-conditioned room (~24 h).

After pre-drying, the tape sheet was sufficiently strong to cut pieces with a diameter of approximately 28 mm from the electrolyte substrate. After the drying process, the thickness of the tape was measured to be 0.7 mm. Cylindrical pieces with a diameter of approximately 28 mm were then cut from the dried ceramic tape.

The ceramic anode tape pieces were subsequently sintered in a Carbolite HTF17/5 chamber furnace in air at temperatures ranging from 1100 °C to 1550 °C for 2 h to prepare the NiO–5CBCY ceramic anode material.

### 2.2. Analytical Methods for Evaluating the Physicochemical Properties of the NiO–5CBCY and Ni–5CBCY Samples

The physicochemical properties of the initial dried NiO–5CBCY ceramic tape were tested, and the sintered NiO–5CBCY anode tape pieces were subjected to the reduction of NiO to metallic Ni. The reduction process for NiO to Ni in the NiO–5CBCY was carried out in a gas mixture containing 10 vol.% H_2_ in Ar using a tube furnace. The sample was then gradually heated to 700 °C, held for 2 h and gradually cooled to RT. The phase composition of the NiO–5CBCY sintered tape before and after the reduction to Ni–5CBCY was determined via X-ray diffraction (XRD) analysis. The microstructures of the NiO–5CBCY and Ni–5CBCY samples were also observed under a scanning electron microscope (SEM, Nova NanoSEM 200, FEI Europe). The pore size distribution in the NiO–5CBCY and Ni–5CBCY samples was examined by mercury porosimetry using a PoreMaster 60 (Quantachrome Instruments). The dried NiO–5CBCY green tape samples were also analysed using thermal analysis. Investigations were conducted using the STA 449 F3 Jupiter apparatus (Netzsch, Germany) as well as the differential thermal analysis (DTA)/thermogravimetry (TG) or differential scanning calorimetry (DSC)/TG technique. These investigations aimed to determine the thermal effects that occurred while the green tape samples were being heated. Other investigations were performed using a thermo-analyser (SDT 2960, TA Instruments) coupled with quadrupole mass spectrometry (QMS), which facilitated an analysis of the gaseous products’ chemical composition while the samples were being heated, particularly in the presence of thermal effects. The TG curves were registered with a TGA/SDTA 851e apparatus (Mettler Toledo). The gas evolution of H_2_O and CO_2_ was analysed online via QMS with a Balzer GSD 300T. A dilatometry apparatus (Netzsch, Germany) was used to identify variations in the dimensions of the samples heated within the range of 25 °C to 1450 °C.

### 2.3. Electrical Measurements of the Ni–5CBCY Samples Using the AC Four-Wire Probe Method and Electrochemical Impedance Spectroscopy

**Figure 1:**
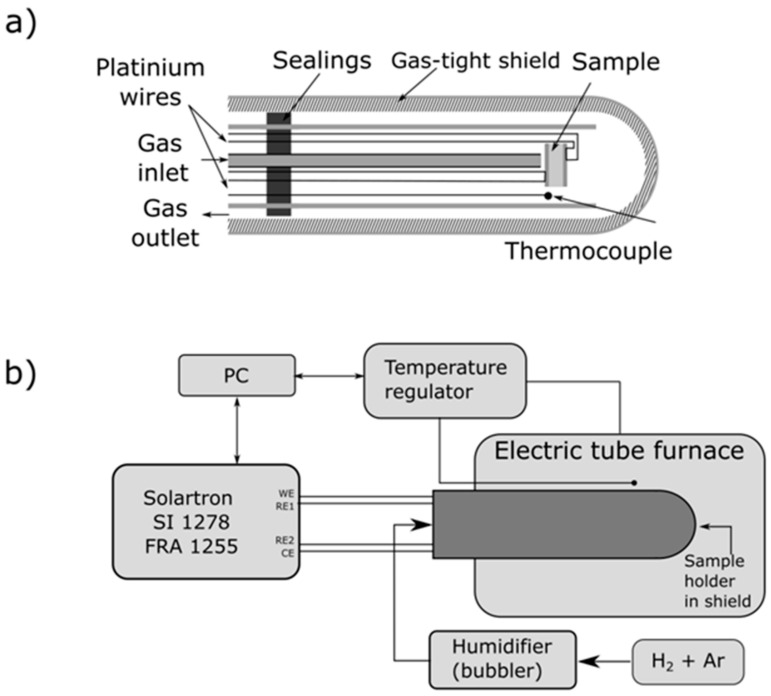
(**a**) Construction of electrical probe for four-wire AC electrical conductivity measurement; (**b**) all of the equipment and apparati used for these experiments.

Total electrical conductivity variations in the Ni–5CBCY samples were determined via electrochemical impedance spectroscopy (EIS). Electrical measurements were performed in the temperature range of 200–750 °C in an H_2_–Ar gas mixture (containing 2–50 vol.% H_2_ in Ar). The spectra were measured at open circuit potential with an AC voltage amplitude of 10 mV beginning from 1 Hz up to 1M Hz with a density of 12 and seven points per decade in the 100 kHz–1 MHz and 1 Hz–100 kHz ranges. A computer program based on the MINUIT [26] software was used to fit the impedance spectra, as previously described [27]. The disc-shaped Ni–5CBCY sample with a thickness of ~0.6 mm and diameter of ~5 mm was mounted between two platinum grids in the gas-tight alumina test apparatus. The construction of the probe for four-wire AC electrical conductivity measurements is illustrated in Figure 1a, while the complete electrical setup with all of the apparatus and equipment is presented in Figure 1b.

The NiO–5CBCY sample was reduced to Ni–5CBCY pellets with a mixture of Ar and H_2_ (10 vol.% H_2_ in Ar) and moistened by bubbling through water at 25 °C with a constant flow rate of 100 cm^3^ min^−1^. For this procedure, the NiO–5CBCY sample was first heated to 700 °C in the holder (Figure 1a,b), and the process of reducing NiO to Ni in the 5CBCY matrix was then carried out. The NiO-to-Ni reduction progress was also monitored using EIS. The Nyquist plots (–*Z*″ vs. *Z*′) were recorded every 10 min for 4 h using a Solartron SI 1255B Frequency Response Analyzer with a 1287 Electrochemical Interface, Zplot 3.3a software and a self-written testing procedure. When the reduction process was complete, the temperature dependence of the impedance was measured sequentially during cooling and heating in the range of 400–700 °C with 5 °C/min^−1^ heating and cooling rates. Measurements of electrical conductivity as a function of temperature were carried out for the Ni–5CBCY cermets in the temperature range of 400–750 °C. Reference measurements of electrical conductivity were also performed for the Ni–5CBCY sample, which had previously been subjected to the reduction process in a separate quartz reactor. In this case, the NiO–5CBCY sample was placed in a platinum crucible and subjected to reduction at 700 °C for 2 h in a gas atmosphere of 10 vol.% H_2_ in Ar.

The dependence of electrical conductivity on temperature was also determined for Ni–5CBCY samples additionally subjected to cyclic Ni-to-NiO oxidation followed by NiO-to-Ni reduction. For this part of the investigation, the samples were oxidised in air at 700 °C for 10 h and then reduced again in the gas mixture of 5 vol.% H_2_ in Ar.

This set of electrical conductivity measurements was repeated in mixtures containing 5% and 10 vol.% H_2_ in Ar for two samples. To examine the impact of the presence of CO_2_ impurity in the hydrogen fuel, the electrical conductivity measurement was performed for Ni–5CBCY cermets using the main 10 vol.% H_2_ in Ar gas mixture with 5 vol.% CO_2_ added to simulate an impurity.

## 3. Results

### 3.1. Optical Microscopy Characterisation of Green NiO–5CBCY Tape Obtained via the Tape Casting Method

**Figure 2:**
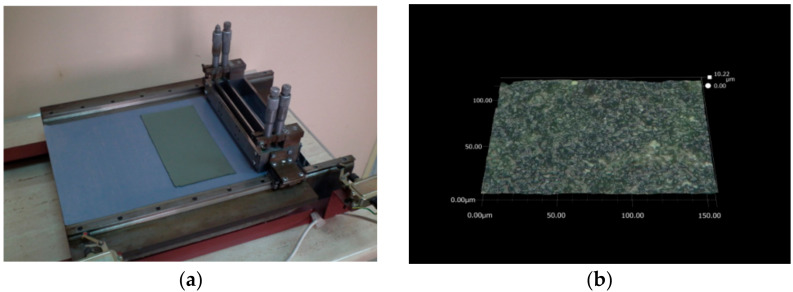
(**a**) Photograph of NiO–Ba_0.95_Ca_0.05_Ce_0.9_Y_0.1_O_3−__δ_ (5CBCY) green anode tape fabricated using the tape casting method; (**b**) surface image of NiO–5CBCY dried anode recorded during optical microscopy investigations.

A photograph of the dried green NiO–5CBCY anode tape on the casting table is shown in Figure 2a.

The dried green tape made from the NiO–5CBCY cermet was 0.7 mm thick. It was flat and flexible and had sufficient mechanical strength to allow it to be easily detached and manipulated to create the desired shapes. It was possible to easily cut circular samples of the NiO–5CBCY (diameter ≈ 28 mm), the physicochemical properties of which were characterised for other analytical purposes. The surface of the dried NiO–5CBCY anode material was inspected using optical microscopy (Figure 2b), revealing the surface to be flat and homogenous. Based on the observations, it can be concluded that the applied experimental conditions for slurry preparation, the ceramic tape casting process and the sheet tape drying conditions resulted in an anode tape sheet satisfying significant quality criteria. For example, no cracks, thickening, or roughness were present on the NiO–5CBCY surface and there were no visible differences in thickness across the entire length of the anode sheet.

**Figure 3:**
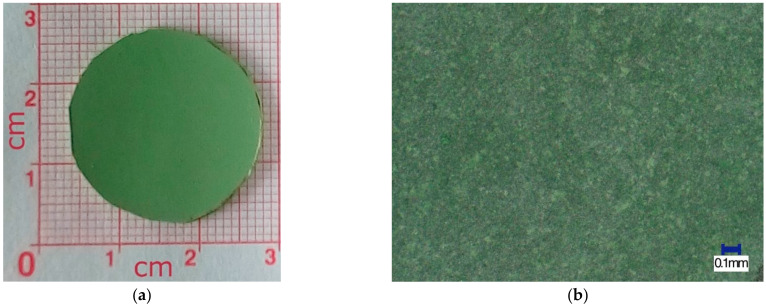
(**a**) Photo of sintered NiO–5CBCY sample; (**b**) optical image of the NiO–5CBCY surface on the sintered cylindrical sample.

The adequate quality of the obtained green tape was confirmed by further investigations of the sintered samples and after the reduction of NiO to Ni in the NiO–5CBCY sample. Figure 3a shows a cylindrical NiO–5BCY sample after sintering, and Figure 3b shows the surface of the NiO–5CBCY anode sample recorded by optical microscopy.

The sintered pieces of the ceramic tape were also found to be free from thickening, beads, cracks, or other surface defects for the porous NiO–5CBCY material before reduction. The tape casting slurry discussed above, which included the ceramic powders NiO and 5CBCY in a non-aqueous organic medium, along with the procedure for film casting and drying, enabled the fabrication of uniform crack-free NiO–5CBCY tape.

### 3.2. Analysis of NiO–5BCY Substrate during Thermal Treatment Using Thermal Analysis Methods

Before the sintering process, a thorough investigation of the thermal behaviour of the green tape was useful, to determine any changes that might occur in the samples during temperature increases along with the impact of temperature on the microstructural quality of the thick film. When the two-phase NiO–5CBCY samples were heated from room temperature (RT) to the final sintering temperature, irreversible variation occurred. Understanding the thermal effects that might occur when the two-phase NiO–5CBCY green tape is heated is highly important in elaborating the optimal conditions for the sintering of dried NiO–5CBCY samples [28,29]. The main requirements for the NiO–5BCY samples as anode supports include not only sufficient mechanical strength and adequate total porosity, but also size distribution before and after the reduction of NiO to Ni in the 5CBCY matrix. Generally, in the construction of anode-supported PCFCs, the surface of NiO–BCY or NiO–BZY is covered by a thin layer of gas-tight proton-conducting electrolyte. Modern fabrication methods for ceramic layers allow for the deposition of a gas-tight thin electrolyte layer (<20 mm) able to uniformly cover the anode surface. Good adhesion is also required in the electrolyte film deposited to the anode surface [30,31,32].

A comprehensive understanding of the thermal effects that occur during temperature increases are enabled by DTA/TG and chemical analyses, facilitating the development of a suitable thermal programme designed to release waste products from the tape (such as organic media, solvents, plasticisers, or pore formers) and ensure its mechanical strength and porosity. The DTA/TG results for 5CBCY electrolyte green tape obtained using the tape casting method are described in [24]. This NiO–5CBCY ceramic tape was manufactured using a very similar organic medium and the same 5CBCY powder, although the two-phase NiO–5CBCY material in the present study contained additional graphite, which is responsible for the porosity developed in the sintered NiO–5CBCY ceramic tape samples.

**Figure 4:**
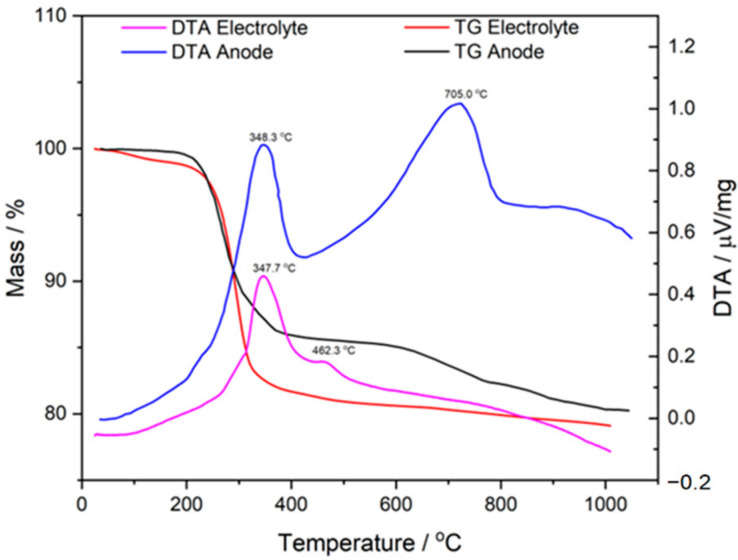
Comparative analysis of differential thermal analysis (DTA)/thermogravimetry (TG) for 5CBCY electrolyte and NiO–5CBCY anode substrate.

Figure 4 presents a juxtaposition of the DTA and TG curves recorded for the 5CBCY and NiO–5CBCY samples while they were heated in air at temperatures ranging from RT to 1100 °C. This juxtaposition aims to compare the variation taking place in materials (one-phase 5CBCY and two-phase NiO–5CBCY) prepared from the same organic carriers and under very similar tape casting conditions. 

The basic difference between the materials is the presence or absence of NiO and graphite as a pore former agent. Based on an analysis of the DTA curves recorded for the 5CBCY and NiO–5CBCY samples, the presence of exothermic effects was determined, with corresponding peaks of varied intensity, at a temperature of ~348 °C (related to the decomposition of the organic medium) and higher temperatures of ~460 °C and ~700 °C (related to the burnout of residual carbon and its release in the form of gaseous products). A detailed TG and DTG analysis in combination with the online measurement of gases via QMS was also performed for the 5CBCY tape, the NiO–5CBCY cermet anode and the cermet anode after sintering. 

**Figure 5:**
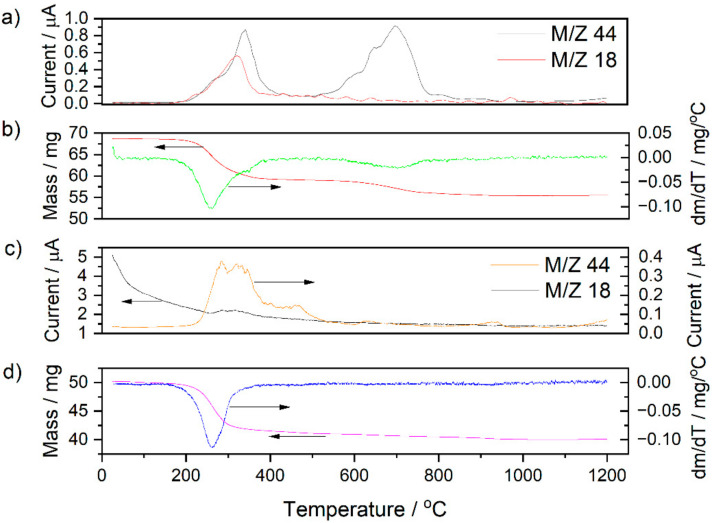
(**a**–**d**) Juxtaposition of recorded variations in the intensity of CO_2_ (M/Z 44) and H_2_O (M/Z 18) in evolving gaseous products during 5CBCY and NiO–5CBCY heating in air.

The TG and DTG curve analysis (Figure 5a–c) recorded within the range studied was consistent with the data presented in the DTA curves. Heating the 5CBCY and NiO–5CBCY samples from RT to 1100 °C led to a gradual loss of mass, with the greatest losses recorded in the temperature range of 200–400 °C for the 5CBCY sample and of 200–300 °C for the NiO–5CBCY sample.

The changes in ionic current reordered for CO_2_ and H_2_O reveal the highest variations were recorded at temperatures between 300 °C and 400 °C, corresponding to the decomposition and burning of organic matter. A QMS analysis was also performed for the sintered non-conductive NiO–5CBCY sample. The evolution of carbon monoxide as an optional product of graphite residue combustion in the residual samples was not observed. The experimental results confirm that the application of thermal treatment is appropriate to obtain NiO–5CBCY as an anode support.

**Figure 6:**
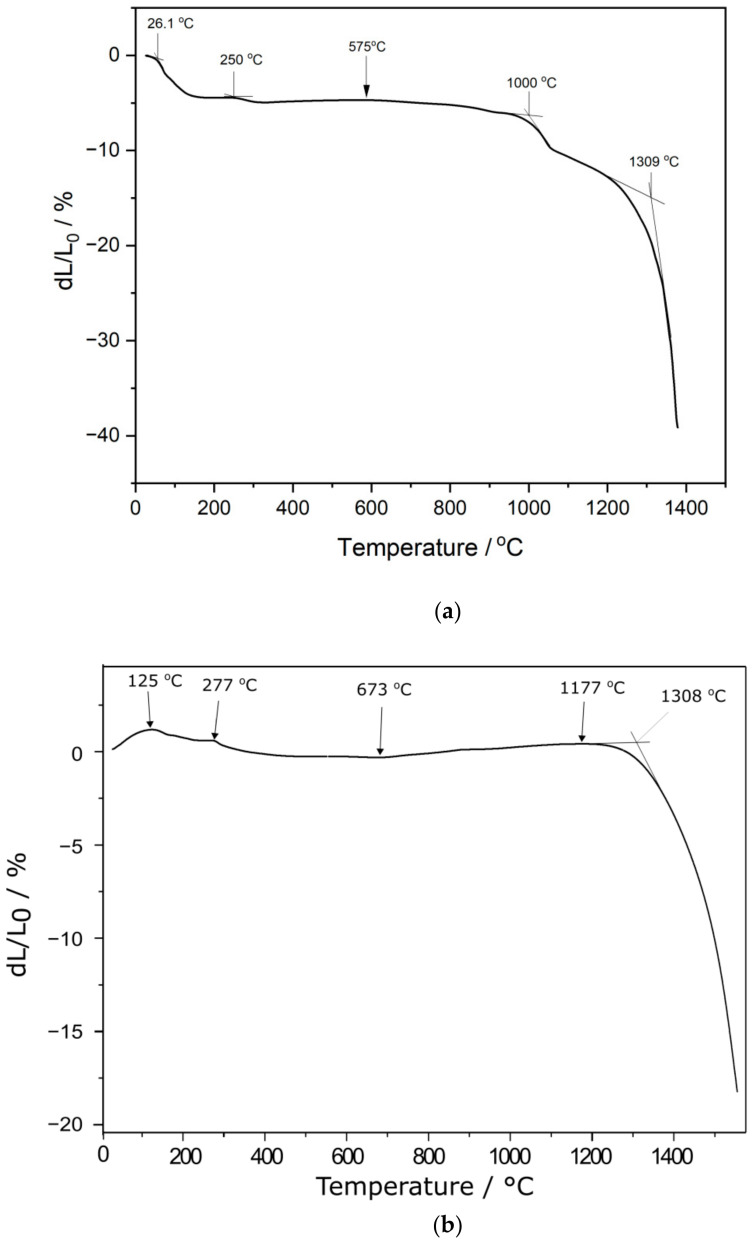
Dilatometric curve recorded for the NiO–5CBCY sample: (**a**) in air; (**b**) in helium.

Dilatometry was applied to study the variation in the dimensions of the dried NiO–5CBCY samples during thermal treatment between 25 °C and 1550 °C. An analysis of the linear shrinkage variation of the NiO–5CBCY anode substrate in relation to temperature was also helpful in establishing the final sintering conditions. Figure 6 shows the dilatometric curves for the NiO–5CBCY sample in air (Figure 6a) and helium (Figure 6b).

Based on the curve shown in Figure 6a, it can be concluded that the first changes in the linear dimensions of the NiO–BC5CY sample took place between 25 °C and 250 °C. These changes were a result of the loss of solvent and the initial decomposition of organic substances. Further, significant changes in the linear dimensions of the sample sintered in air were observed above 1000 °C (Figure 6a). At 1309 °C, intensive linear shrinkage occurred, and at 1400 °C, the shrinkage was approximately 40% of the initial length.

Based on the curve in Figure 6b, it was found that significant changes in the linear dimensions of the NiO–BC5CY sample began when the temperature reached ~1300 °C. Further heating led to a gradual increase in shrinkage, with the value reaching 29.5% at ~1400 °C. A comparative analysis of the curves presented in Figure 6a,b reveals that the shrinkage of the NiO–BC5CY sample reached lower values across the entire tested temperature range in helium than in air. The reduced shrinkage in helium flow was likely due to the absence of carbon and graphite burning, substances which were introduced during the preparation of the suspension or formed during the decomposition of the organic components of the slurry. The removal of residual carbon during the sintering of the NiO–5CBCY samples was also verified through chemical analysis of the evolved gases using the QMS method. The sintered NiO–5CBCY samples were heated to temperatures ranging from 25 °C to 1200 °C, and TG and QMS curves were recorded under these conditions. No additional CO_2_ current was observed in the sample QMS curve. Based on DTA/TG and dilatometric analysis, the gradual sintering programme was expanded.

**Figure 7:**
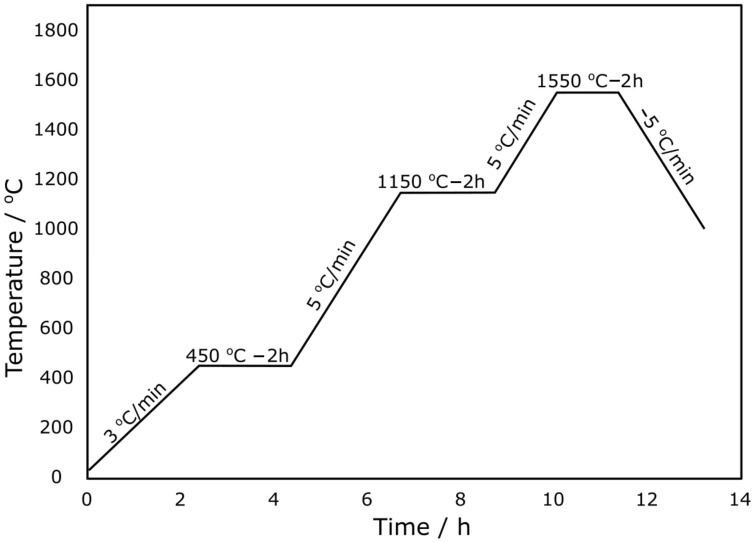
Elaborated profile of temperature increases while sintering the NiO–5CBCY anode sample.

Figure 7 presents the gradual sintering programme for the NiO–5CBCY sample used to obtain the porous material. In the first stage of this process, the anode carrier was slowly heated at a rate of 3 °C/min from 25 °C to 450 °C. The purpose of this stage was to remove water and initiate decomposition of the organic medium used during cermet foil formation. In the second stage (i.e., at >450 °C), the sample was conditioned for 2 h with the aim of slowly removing the organic compounds used during ceramic foil formation without introducing cracking or other deformations. In the third stage, the anode carrier was gradually heated from 450 °C to 1150 °C to remove residual carbon originating from the decomposition of organic compounds. Under these conditions, the gradual oxidation of graphite could occur, and the gradual increase in temperature caused further oxidation/combustion in the atmosphere. The largest variations in the linear dimensions of the sample over time were observed from 1050 °C to 1450 °C due to the initiation of the sintering process. Eventually, a final sintering temperature of 1550 °C was adopted, and the total sample sintering time was 2 h.

**Figure 8:**
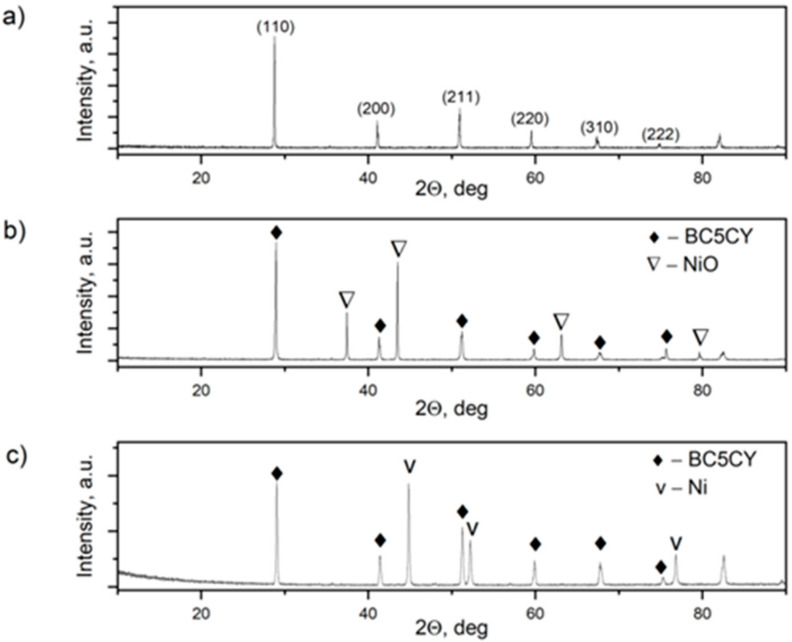
X-ray diffraction (XRD) patterns recorded for: (**a**) 5CBCY; (**b**) NiO–5CBCY anode substrate; (**c**) Ni–5CBCY anode support.

Identification of the phase composition of the NiO–5CBCY samples before and after reduction was enabled by XRD analysis. Figure 8a–c presents the recorded XRD patterns for, respectively, the original 5CBCY powder, the NiO–5CBCY anode substrate sintered at 1550 °C for 2 h and the Ni–5CBCY sample following reduction at 700 °C for 2 h in 10 vol.% H_2_ in Ar.

Based on Figure 8, it can be concluded that the desired phases of NiO and 5CBCY perovskite can be obtained by sintering NiO–5CBCY tape as a substrate for anode support at 1550 °C for 2 h according to the thermal treatment programme outlined above. Additionally, further heating the NiO–5CBCY sample at 700 °C for 2 h in the gas atmosphere of 10 vol.% H_2_ in Ar reduced NiO to Ni without changing the phase composition of the second component of the 5CBCY cermet. The Ni–5CBCY anode support can thus be successfully prepared by this method.

### 3.3. Scanning Electron Microscopy with Energy Dispersive X-ray Spectroscopy of the Ni–5CBCY Sample Microstructure

**Figure 9:**
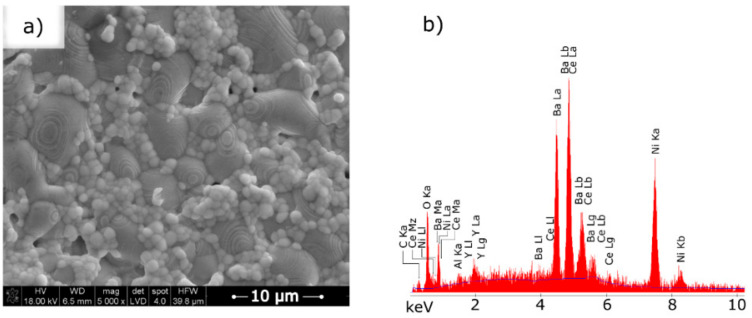
(**a**) Scanning electron microscopy (SEM) micrograph of the surface of the anode substrate sintered at 1550 °C for 2 h; (**b**) EDX spectra of the sintered NiO–5CBCY samples.

Figure 9a presents an SEM micrograph of the sintered NiO–5CBCY anode tape.

The observed NiO–5CBCY sample was flat, and the NiO and 5CBCY electrolyte grains were evenly distributed. There were several visible pores on the sample surface, likely formed during the sintering of the graphite grains introduced into the casting slurry. In the obtained NiO–5CBCY composite material, the NiO grain size ranged from 5 to 8 µm, while the BC5CY electrolyte grain size ranged from 1 to 2 µm.

**Figure 10:**
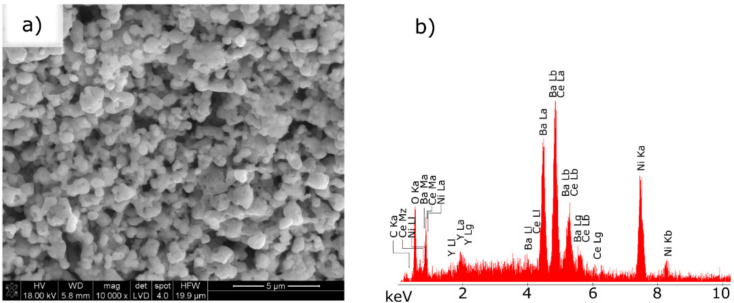
(**a**) Evolution of microstructure in the sintered NiO–5CBCY sample resulting from the reduction of NiO to Ni and thermal treatment at 700 °C for 2 h in 10 vol.% H_2_ in Ar (**b**) EDX spectra of the reduced Ni–5CBCY samples.

**Figure 11:**
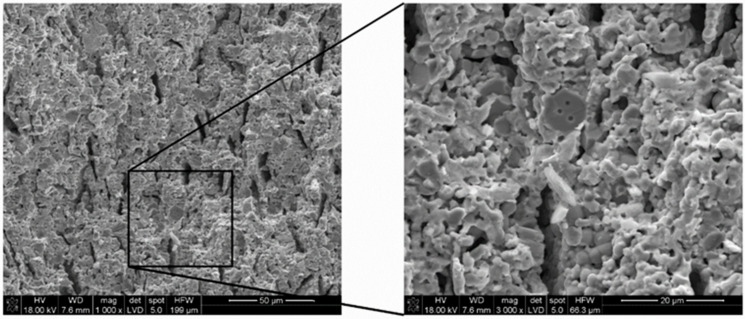
SEM micrograph showing a cross section of the reduced Ni–5CBCY anode substrate.

In the second stage, the microstructure of the Ni–5CBCY anode substrate was observed when subjected to reduction by a gas atmosphere of 5 vol.% H_2_ in Ar (Figure 10a,b and Figure 11).

Based on the recorded SEM micrographs, it can be concluded that the reduction of NiO to metallic Ni in the 5CBCY matrix led to increased porosity compared with the sample before the reduction process. Figure 11 presents a cross-sectional analysis of the obtained conductive Ni–5CBCY anode.

Figure 11 shows that the cross section of the Ni–5CBCY sample exhibited uniform porosity. Mercury porosimetry was applied to study the pore size distribution following the reduction process. The samples were reduced in 10 wt.% H_2_ in Ar.

To analyse the impact of the experimental conditions on the variation in pore size distribution in the Ni–5CBCY samples, the temperature was increased from RT to 750 °C, and different heating rates were applied (2.5 °C/min, 5.0 °C/min and 10 °C/min).

**Figure 12:**
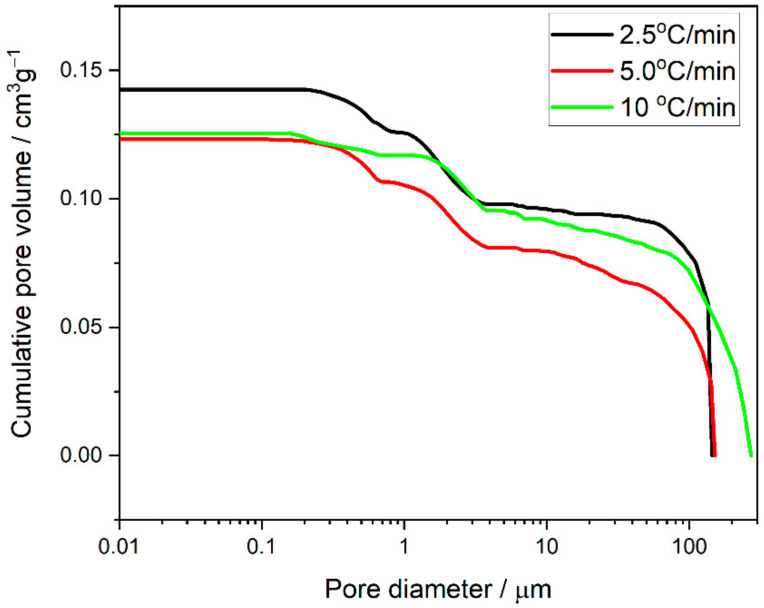
Cumulative pore volume vs. pore diameter recorded for the Ni–5CBCY samples.

From Figure 12, it can be concluded that, apart from technological factors (the chemical composition and physicochemical properties of the suspension), the factors influencing the pore size and distribution included the conditions for reducing NiO to metallic Ni.

The pore size distributions of all the materials were similar, with three distinct populations of pores. The largest pores had diameters between ~50 and 250 μm and formed ~60% (for 2.5 °C/min and 5 °C/min) to 70% (for 10 °C/min) of the open porosity of the material. In both the former materials, the population of the largest pores was relatively small compared with that of the material annealed at 10 °C/min. The two other populations of pores were 1–4 μm and 0.1–1 μm and were almost identical at 2.5 °C/min and 5.0 °C/min. The results might indicate that, under hydrogen flow, the relatively low rate of NiO-to-Ni reduction did not influence the smallest pores in the Ni–5CBCY sample. It should be emphasised that the problem of microstructure optimisation for the Ni–BCY anode support has not yet been fully understood or explained [33,34].

Nickel–yttria-stabilised zirconia (Ni–YSZ) is a well-known anode support for SOFCs, with an oxide ion-conducting O_2_ construction. When Ni–YSZ anodes are used for SOFCs, a high porosity (55 wt.%) is necessary to achieve high performance. In previous works [35,36], researchers investigated the impact of total porosity on an Ni–YSZ anode support. In the samples, the anode porosity varied from 32% to 76% in four test cells, and the peak power density varied from 0.72 W/cm^2^ (for a porosity of 32%) to 1.55 W/cm^2^ (for a porosity of 57%). In short, the increase in porosity significantly enhanced the performance. At the same time, in [16,37], the authors announced that an open porosity of ~48 vol.% in Ni–YSZ samples was suitable for ensuring gas permeability in the anode materials. In contrast to Ni–YSZ, the optimal ratio of Ni:BCY or NI:BZY in Ni–BCY or Ni–BZY ceramic anodes has not yet been determined. Similar challenges regarding the porosity for these materials also exist. Data in the existing literature indicate that in PCFCs, sufficient electrical conductivity can be maintained at a lower total porosity (25–35 vol.%) [38,39].

The total electrical conductivity (σ) of anode materials is the primary criterion used to select fuel electrodes for application in SOFCs. While it has been concluded that the minimum σ value should be ~1 S/cm, increasing it above 10 S/cm appears to be superior for such applications. In practical applications, it is also important to have stable electrical conductivity during operation over long periods as well as during thermal and redox cycling upon startup and shutdown. The σ-dependence of Ni–5CBCY samples in relation to temperature was determined using EIS measurements in a reduced atmosphere (10 vol.% H_2_ in Ar).

Table 2 lists the data for electrical measurements performed for Ni–5CBCY in 10 vol.% H_2_ in Ar. It also lists the electrical conductivity values for Ni–BCY and Ni–BZY anode materials at 700 °C.

Based on the data in Table 2, it can be concluded that the Ni–5CBCY anode material obtained in this work had a lower electrical conductivity than Ni–BCY cermet. There are a number of possible reasons for this difference. In a previous work, we found that 5CBCY electrolyte is characterised by a lower electrical conductivity compared with BCY. The data existing in the literature on BCY also indicate that the electrical conductivity of BCY is strongly dependent on the microstructure and sintering conditions of the gas-tight samples. It is well known that the electrical resistance of the grain boundaries in sintered BCY samples has a considerable impact on the total electrical resistance of the samples. In particular, resulting differences in the total electrical conductivity of more than half an order of magnitude can be observed. The main advantage of 5CBCY compared with BCY is its higher chemical resistance to CO_2_ [8,9]. It should be emphasised that the problem resulting from changes in electrical conductivity as well as the selection of reference quantities are more complex in anode materials containing Ni and a ceramic proton conductor than in cermet anodes containing metallic Ni in a matrix of YSZ or GDC solid solution, which are known conductors of O^2−^ ions. In addition to the chemical composition of the anode material (i.e., the share of metallic Ni particles dispersed in the ceramic ion conductor matrix), the microstructure also plays a crucial role in the degree of electrical conductivity and electrocatalytic activity. Specifically, the relevant variables include the dispersion of Ni particles in the matrix of the ionic conductor and the proportion and distribution of the pore size in the target anode material. The elaboration of fabrication methods for NIO–5CBCY powders by wet chemical methods, the selection of adequate carbon-based pore formers to create porosity and the tailoring of the microstructure by optimizing the sintering conditions can also lead to improvements in the electrical conductivity and electrochemical activity for fuel oxidation.

**Figure 13:**
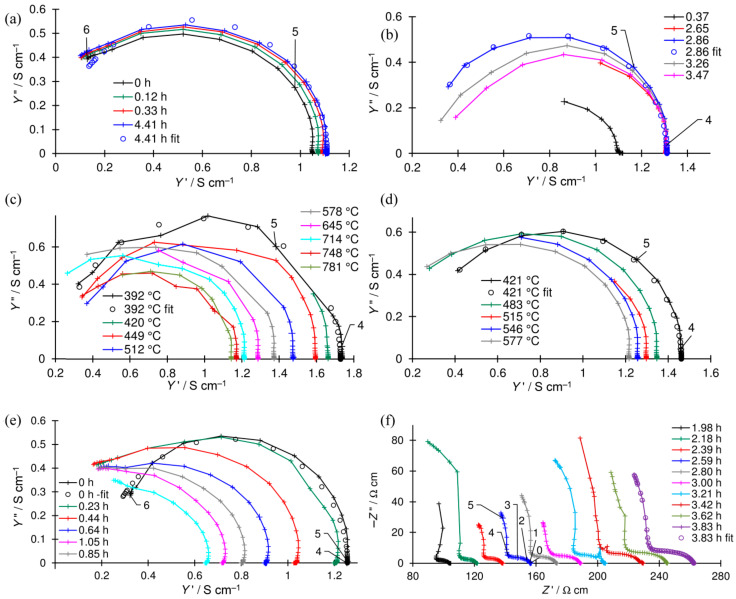
Examples of admittance (**a**–**e**) and impedance spectra (**f**) during: (**a**) the first and (**b**) second reductions in 5% H_2_; (**c**) heating and (**d**) cooling; during oxidation in wet air (**e**) before and (**f**) after the loss of metallic conductivity (crosses—measured data, circles—fitted data). The numbers above the magnified symbols represent the logarithm of the frequency. In (**a**,**b**,**e**,**f**), the legend shows the time elapsed from the beginning of the measurement. In (**c**,**d**), it shows the temperature.

Here, EIS was also used to determine the electrical properties, with the measurements performed on a reduced Ni–5CBCY sample. Examples of admittance and impedance spectra are presented in Figure 13a–f, respectively.

**Figure 14:**
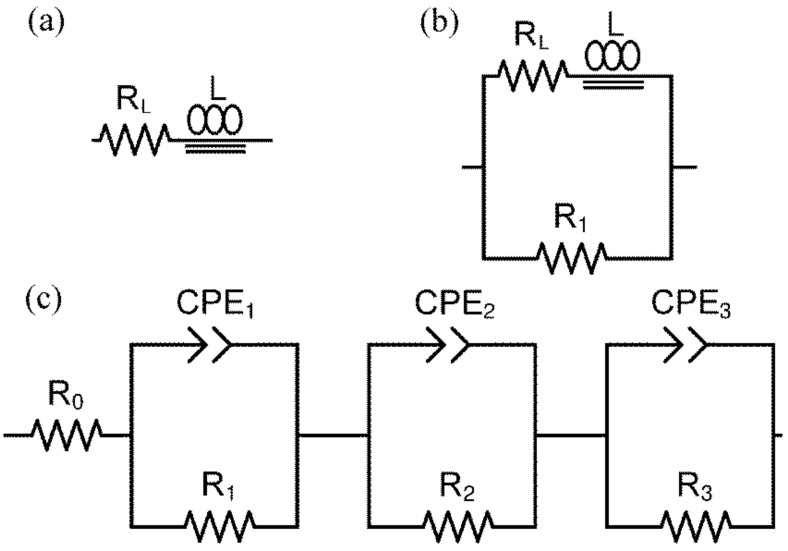
Electrical equivalent circuits used to fit the impedance spectra: (**a**,**b**) Reduced samples; (**c**) oxidised samples.

On the spectra presented in Figure 13a–e, a single inductive high-frequency (HF) semicircle representing the conductivity of the sample is shown. The right-hand-side crossing point of the semicircle with the abscissa is attributed to the value of the total admittance [42]. The changes in the sample’s conductivity can be evaluated graphically or based on the results of fitting. Depending on the shape of the spectrum, one of the electrical equivalent circuits (EECs) shown in Figure 14a,b was selected. These EECs consist, respectively, of a serial-connected inductor L and resistor R_L_ (Figure 14a) and an inductor L, resistor R_L_ and parallel-connected resistor R_1_ (Figure 14b) [43,44].

The shape of the spectrum presented in Figure 13f consists of one large HF and two small overlapping low-frequency (LF) capacitive semicircles. The total resistivity of the sample can be evaluated graphically as the intersection of the HF semicircle at the LF side with the abscissa axis. The EEC used to fit the impedance spectra recorded for the oxidised sample, consisting of three overlapped semicircles (as previously described in [45]), is presented in Figure 14c. It consists of a resistor, R_0_, which represents the bulk resistance not visible in the spectra due to the applied frequency range, and three subcircuits connected in series. Each subcircuit consists of a parallel-connected resistor and constant phase element (CPE). The first (R, CPE) subcircuit represents the grain boundary impedance of the sample, whereas the second and third represent the electrode reaction. Thus, the resistance of the sample is the sum of *R*_0_ and *R*_1_.

The fitting results are collated in Table 3, Table 4, Table 5, Table 6, Table 7 and Table 8. In the first column, the time elapsed at the beginning of the experiment (Table 3, Table 4, Table 7 and Table 8) or temperature (Table 5 and Table 6) is given. In the second column, the standard deviation characterising the quality of the fit is given. The standard deviation is calculated using the following formula [27]:

(1) $$s=\sqrt{\frac{\sum_{1}^{n}{\left(\frac{\mathrm{Modulus}\left(Z_{i,measured}-Z_{i,fitted}\right)}{\mathrm{Modulus}\left(Z_{i,measured}\right)}\right)}^{2}}{n-1}},$$

where *Z_i_* is the impedance at the frequency number *i*, and *n* is the number of frequencies in the impedance spectrum. The next columns contain the fitting results. Capacitances are given instead of CPE parameters, calculated using the following formula:

(2) $$C_{i}=\frac{1}{2\pi f_{0}Z_{{\mathrm{CPE}}_{i}}}{\left(\frac{f_{0}}{jf}\right)}^{\alpha }$$

where *f* is the frequency, *f*_0_ is the reference frequency assumed to be *f*_0_ = 1000 Hz, j is the imaginary unit, *C*_i_ is the capacitance at the reference frequency, and the index i is the CPE number (Figure 14a). In the last column, the electrical conductivity of the sample is calculated using the following formula:

(3) $$\sigma =\frac{1}{R_{0}+R_{1}}$$

for spectra fitted using the CPE presented in Figure 14c (Table 8):

(4) $$\sigma =\frac{1}{R_{L}}$$

for spectra fitted using the CPE presented in Figure 14a (Table 3 and Table 7), and:

(5) $$\sigma =\frac{1}{R_{L}}+\frac{1}{R_{1}}$$

for spectra fitted using the CPE presented in Figure 14b (Table 4, Table 5 and Table 6).

**Figure 15:**
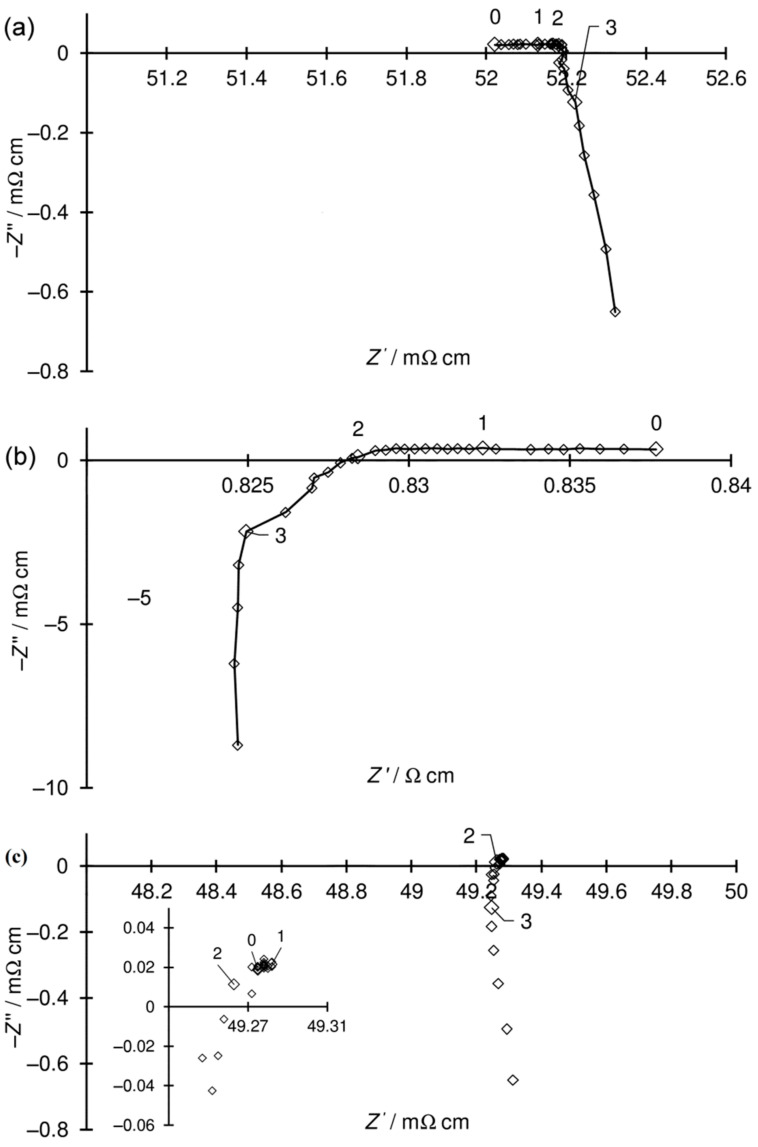
Examples of EIS spectra: (**a**) during reduction (decrease in impedance is visible); (**b**) during oxidation (increase in impedance is visible); (**c**) reduced sample (inset shows the enlarged capacitive part of the spectra).

The system was not in a steady state during the reduction or oxidation of the sample (Figure 15a,b, respectively). Accordingly, the corresponding spectra were not analysed using the methods derived for equilibrium or stationary states. In these cases, the intercept of the spectra with the real axis of the Nyquist (Z′, Z″) plot was used to evaluate the resistance [46,47].

**Figure 16:**
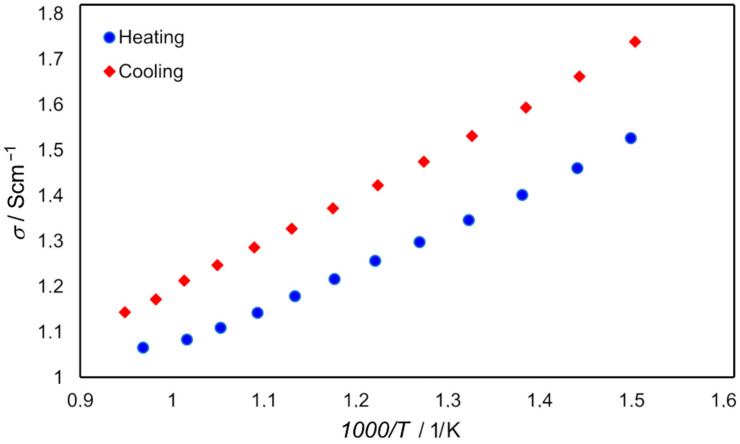
Electrical conductivity (σ) of the reduced Ni–5CBCY sample vs. temperature.

**Figure 17:**
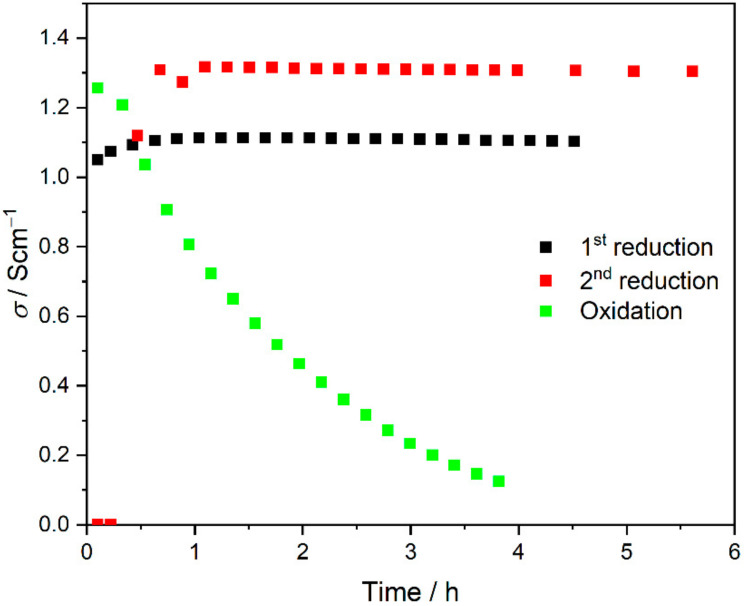
Electrical conductivity (σ) of the Ni–5CBCY reduced samples (black and red) and the sample undergoing oxidation (green) over time. All measurements were performed at 700 °C.

The other almost stable spectrum alongside the inductive loop represents the capacitive component, which was very small and noisy in the reduced sample spectra (Figure 15c), whereas two quite large capacitive components were in the oxidised sample spectra (Figure 13f). The electrical conductivity of the reduced Ni–5CBCY sample in-creased with decreasing temperature, demonstrating that the majority of the conductivity was the electronic conductivity associated with the existence of the well-contacted metallic Ni particle (Figure 16). The electrical conductivity of the fully reduced sample was stable over time and equal to 1.11 and 1.31 S cm^−1^ after the first and second reductions, respectively. During the oxidation process, the electrical conductivity of the sample measured at 700 °C decreased over time and dropped sharply after 220 min, which is related to the loss of continuity of the Ni particles and consequent loss of electronic conductivity (Figure 17) during the oxidation of Ni-to-NiO.

Figure 16 presents the dependence of electrical conductivity (*σ*) on temperature for the heating and cooling cycle.

A slight variation in the electrical conductivity of the Ni–5CBCY reduced sample in relation to temperature was observed, demonstrating that most of the electrical conductivity was electronic conductivity associated with the existence of well-contacted metallic Ni particles. During the cooling cycle, the electrical conductivity should be slightly improved due to a possible increase in the humidity of the Ni–5CBCY samples. A performance assessment of metallic Ni in the anode cermets should also consider the redox operation. The Ni-to-NiO transition in the 5CBCY matrix oxidation process not only causes bulk volume expansion but also variation in the Ni structure. For its part, the reduction of NiO-to-Ni causes material shrinkage and induces an increase in porosity [48,49,50]. In the oxidised NiO–5CBCY sample, the electrical conductivity decreased over time and dropped sharply after 220 min as a result of NiO formation (Figure 17).

Figure 17 shows the achievement of stable electrical conductivity values close to those observed before oxidation in the Ni–5CBCY sample subjected to Ni-to-NiO oxidation in the 5CBCY matrix and then to NiO-to-Ni reduction. As the results demonstrate, the Ni–5CBCY samples have repeatable electrical conductivity in their reduction and oxidation cycles.

The electrical conductivity of the Ni–5CBCY cermet was also measured using the AC four-probe method in the same H_2_–Ar gas atmosphere but with an additional 5 vol.% CO_2_. The electrical conductivity recorded for Ni–5CBCY at 700 °C for 12 h was 1.1 S/c (10 vol.% H_2_ in Ar), while the value for Ni–5CBCY was slightly lower after the same duration at 0.8 S/cm in 10 vol.% H_2_ in Ar with 5 vol.% CO_2_. Similar measurements were performed for Ni–BCY with σ values of ~2.1 (S/cm). However, for the electrical measurements performed in the 10 vol.% H_2_ in Ar gas atmosphere with an additional 5 vol.% CO_2_, the conductivity decreased to 1.1 S/cm after 12 h at 700 °C.

## 4. Conclusions

This paper presents the results of preliminary research into the possibility of producing Ni–5CBCY cermet anodes with increased chemical resistance to CO_2_ for use in ceramic fuel cells containing a proton conductor as an electrolyte. The application of slurry prepared from NiO, 5CBCY, graphite and organic compounds yielded good-quality tape sheets. Optical microscopic examinations revealed the obtained dried ceramic tapes had no surface or structural defects (such as cracks, loss, or delamination) that would limit the validity of further research. The elaborated sintering curve of the cylindrical NiO–5CBCY sample allowed for the obtainment of porous flat-parallel ceramic tapes with good mechanical strength. Analysis of the chemical composition of the gases released during the thermal treatment of the sintered samples confirmed the complete removal of all graphite during the applied anode substrate burning process. Phase composition tests using XRD analysis on sintered NiO–5CBCY tape and on sintered samples subjected to NiO reduction to Ni–5CBCY in a gas atmosphere of 10 vol.% H_2_ in Ar were also undertaken. The results confirmed the receipt of the desired two-phase NiO and perovskite 5CBCY for ceramic sintered tapes and metallic Ni as well as Ba_0.95_Ca_0.05_Ce_0.95_Y_0.1_O_3_ after heating the samples in an Ar–H_2_ gas atmosphere.

The electrical conductivity of the Ni–5CBCY samples is sufficient for the preliminary testing of the obtained materials in PCFCs. Electrical conductivity measurements found that the Ni–5CBCY cermet exhibited repeatable electrical conductivity values after Ni-to-NiO oxidation cycles and NiO-to-Ni reduction in the 5CBCY matrix. The addition of 5 vol.% CO_2_ as an impurity into the H_2_–Ar gas mixture led to a greater decrease in the electrical conductivity of the Ni–5CBCY cermet compared with the original Ni–BCY sample under the same conditions.

## Figures and Tables

**Table 1:** Juxtaposition of the chemical reagents used to fabricate the Ni–5CBCY anode.

Component	Mass/g
BC5CY electrolyte powder	38
Nickel (II) oxide	57
Graphite	5
Organic binder PVB, plasticiser, solvent	85

**Table 2:** Comparison of the electrical conductivity (σ) of Ni–5CBCY; Ni-BCY and Ni-BZY anode materials at 700 °C.

No	σ/S cm^−1^	Anode Material/Conditions	Ref.
1	1.1	Ni–5CBCY (57 wt% NiO) Humidified 10 vol.% H_2_ in Ar	This work
2	~3.1	Humidified 10 vol.% H_2_ Ni–BZY—50 vol.% Ni BZY = BaZr_0.85_Y_0.15_O_3−d_	[40]
3	~40	Ni–BCY (40–60 wt% NiO) Humidified H_2_	[41]

**Table 3:** Results of fitting the impedance spectra recorded for Ni–5CBCY during the first reduction in 5% H_2_–Ar gas atmosphere (*t*—time elapsed from the beginning of the experiment; *s*—standard deviation; fitted parameters (for the electrical equivalent circuit (EEC) presented in Figure 14a): *L*—inductance; *R*_L_—resistance; σ—electrical conductivity).

*t*/h	*s*/%	*L*/mH	*R*_L_/W cm	σ/S cm^−1^
0.00	1.06	0.410	0.95	1.05
0.12	0.88	0.412	0.93	1.07
0.33	0.88	0.410	0.91	1.09
0.53	0.90	0.409	0.90	1.11
0.73	0.91	0.408	0.90	1.11
0.94	0.91	0.407	0.90	1.11
1.14	0.90	0.407	0.90	1.11
1.35	0.91	0.406	0.90	1.11
1.55	0.90	0.405	0.90	1.11
1.76	0.91	0.405	0.90	1.11
1.96	0.92	0.405	0.90	1.11
2.17	0.89	0.405	0.90	1.11
2.37	1.08	0.406	0.90	1.11
2.57	0.91	0.405	0.90	1.11
2.78	0.68	0.409	0.90	1.11
2.98	0.89	0.405	0.90	1.11
3.19	0.81	0.407	0.90	1.11
3.39	0.90	0.405	0.90	1.11
3.60	0.91	0.405	0.90	1.11
3.80	0.91	0.405	0.90	1.11
4.01	0.89	0.405	0.90	1.11
4.21	0.91	0.404	0.91	1.10
4.41	0.91	0.405	0.91	1.10

**Table 4:** Results of fitting the impedance spectra recorded during the second reduction in 5% H_2_–Ar gas atmosphere (*t*—time elapsed from the beginning of the experiment; *s*—standard deviation; fitted parameters (for the EEC presented in Figure 14b): *L*—inductance; *R*_L_ and *R*_1_—resistances; σ—electrical conductivity).

*t*/h	*s*/%	*R*_1_/W cm	*L*/nH	*R*_L_/W cm	σ/S cm^−1^
0.37	2.88	1.55	0.011	2.10	1.12
0.58	0.38	8.56	0.575	0.84	1.31
0.99	1.75	1.58	9.21	1.46	1.32
1.20	1.41	3.73	51.63	0.96	1.31
1.41	3.06	1.39	0.91	1.68	1.31
1.62	0.79	1.73	0.228	1.36	1.31
1.82	2.37	3.09	1.00	1.01	1.31
2.03	1.50	1.72	0.99	1.37	1.31
2.23	1.53	1.12	0.58	2.39	1.31
2.23	2.86	3.04	0.70	1.03	1.30
2.44	0.60	1.98	0.011	1.24	1.31
2.65	1.22	2.15	0.060	1.18	1.31
2.86	0.84	3.73	0.012	0.96	1.31
3.06	0.97	3.62	0.68	0.97	1.31
3.26	5.03	2.86	0.90	1.04	1.31
3.47	4.93	2.38	1.06	1.13	1.31
3.68	2.92	1.52	1.60	1.55	1.30
3.89	2.03	3.21	0.76	1.00	1.31
4.43	0.79	3.06	0.710	1.02	1.31
4.97	0.53	5.36	0.542	0.89	1.31

**Table 5:** Results of fitting the impedance spectra recorded for Ni–5CBCY samples at different temperatures during heating (*T*—temperature; *s*—standard deviation; fitted parameters (for the EEC presented in Figure 14b): *L*—inductance; *R*_L_ and *R*_1_—resistances; σ—electrical conductivity).

*T*/°C	*s*/%	*R*_1_/W cm	*L*/mH	*R*_L_/W cm	σ/S cm^−1^
392	2.96	4.49	0.53	0.66	1.73
420	1.81	3.56	0.59	0.72	1.67
449	1.62	3.86	0.60	0.75	1.60
481	2.09	2.02	0.93	0.96	1.53
512	2.70	3.39	0.65	0.85	1.47
544	2.10	24.18	0.41	0.73	1.42
578	1.59	11.28	0.45	0.78	1.37
612	0.24	14.76	0.42	0.79	1.33
645	2.08	5.60	0.54	0.90	1.28
680	1.76	18.54	0.41	0.84	1.25
714	1.47	18.37	0.41	0.86	1.21
745	1.45	3.15	0.77	1.17	1.17
748	1.71	4.60	0.59	1.05	1.17
781	1.73	5.00	0.58	1.06	1.15

**Table 6:** Results of fitting the impedance spectra recorded at different temperatures during cooling (*T*—temperature; *s*—standard deviation; fitted parameters (for the EEC presented in Figure 14b): *L*—inductance; *R*_L_ and *R*_1_—resistances; σ—electric conductivity).

*T*/°C	*s*/%	*R*_1_/W cm	*L*/mH	*R*_L_/W cm	σ/S cm^−1^
394	0.73	3.77	0.64	0.79	1.53
421	0.61	4.00	0.62	0.83	1.46
451	1.88	2.96	0.79	0.94	1.40
483	1.24	8.22	0.49	0.82	1.35
515	0.31	4.93	0.56	0.91	1.30
546	0.43	10.24	0.49	0.86	1.26
577	1.17	12.71	0.45	0.88	1.22
609	1.13	10.35	0.47	0.92	1.18
642	0.40	4.96	0.54	1.06	1.14
677	0.58	3.67	0.71	1.19	1.11
711	3.45	14.64	0.39	0.99	1.08
760	2.41	6.15	0.53	1.11	1.07

**Table 7:** Results of fitting the impedance spectra recorded for Ni–5CBCY during oxidation in wet air before the loss of metallic conductivity (*t*—time elapsed from the beginning of the experiment; *s*—standard deviation; fitted parameters (for the EEC presented in Figure 14a): *L*—inductance; *R*_L_—resistance; *σ*—electrical conductivity).

*t*/h	*s*/%	*L*/mH	*R*_L_/W cm	σ/S cm^−1^
0.00	0.88	0.406	0.797	1.25
0.23	1.33	0.397	0.83	1.20
0.44	1.33	0.379	0.97	1.03
0.64	1.36	0.371	1.10	0.91
0.85	0.89	0.361	1.24	0.806
1.05	0.86	0.356	1.38	0.724
1.25	1.00	0.336	1.54	0.651
1.46	0.95	0.329	1.72	0.580
1.66	1.59	0.298	1.94	0.517
1.85	1.81	0.340	2.17	0.460

**Table 8:** Results of fitting the impedance spectra recorded for the Ni–5CBCY sample during oxidation in wet air and after the loss of metallic conductivity (*T*—temperature; *s*—standard deviation; fitted parameters (for the EEC presented in Figure 14c): *L*—inductance; *R*_0_, *R*_1_, *R*_2_, *R*_3_—resistances; *C*_1_, *C*_2_, *C*_3_—capacitances; *α*_1_, *α*_2_, *α*_3_—coefficients; σ—electric conductivity).

*t*/h	*s*/%	*R*_0_/Ω cm	*R*_1_/Ω cm	*R*_2_/Ω cm	*R*_3_/Ω cm	*C*_1_/pF cm^−1^	*C*_2_/μF cm^−1^	*C*_3_/μF cm^−1^	*α*_1_	*α*_2_	*α*_3_	σ/mS cm^−1^
1.98	3.85	0.0	97.3	6.0	1.22	393	12.7	1510	1.00	0.74	0.67	10.3
2.18	4.89	0.0	113.0	5.3	3.15	235	4.95	107	1.20	1.00	0.87	8.85
2.39	0.5	0.0	123.8	4.49	10.0	153	2.70	7.55	1.08	0.61	0.59	8.08
2.59	0.17	0.0	139.4	5.61	11.7	135	2.04	7.25	1.10	0.74	0.58	7.17
2.8	0.16	0.0	154.2	7.09	12.3	119	1.40	7.32	1.13	0.78	0.61	6.49
3	0.19	0.0	166.4	10.3	12.4	137	1.99	6.62	1.01	0.60	0.55	6.01
3.21	0.57	14.8	166.9	7.70	15.4	122	1.25	6.53	1.16	0.81	0.64	5.50
3.42	2.38	4.78	190.4	31.0	3.25	80	1.17	88	1.15	0.53	0.76	5.12
3.62	0.51	15.2	198.5	21.6	9.82	104	1.09	13.0	1.11	0.62	0.70	4.68
3.83	0.12	10.1	218.2	22.14	12.0	93.5	0.89	10.30	1.10	0.65	0.70	4.38

## Data Availability

Does not apply to this work.

## Abbreviations

PCFCs	ceramic proton-conducting fuel cells
SOFCs	solid oxide fuel cells
A-SOFCs	anode-supported solid oxide fuel cells
BCY	BaCe_0.9_Y_0.1_O_3−δ_, barium cerate-doped yttria oxide, electrolyte
5CBCY	Ba_0.95_Ca_0.05_Ce_0.9_Y_0.1_O_3−δ_, co-doped calcia and yttria barium cerate
NiO–5CBCY	NiO-Ba_0.95_Ca_0.05_Ce_0.9_Y_0.1_O_3−δ_: sintered anode samples
Ni-5CBCY	Ni-Ba_0.95_Ca_0.05_Ce_0.95_Y_0.1_O_3−δ_: cermet obtained by thermally treating
NiO–5CBCY	in H_2_-Ar gas atmosphere at 700 °C for 2 h
DTA	differential thermal analysis
TG	thermogravimetry
QMS	quadrupole mass spectrometry
SEM	scanning electron microscopy
EPD	electrophoretic deposition layers
LSCF	La_0.8_Sr_0.2_Co_0.8_Fe_0.2_O_3_, cathode
YbBC	BaCe_0.9_Yb_0.1_O_3_, barium cerate-doped ytterbium oxide
σ	total electrical conductivity
RT	room temperature
HF	high-frequency
LF	low-frequency
EEC	electrical equivalent circuit

## References

[1] Rasaki S.A., Liu C., Lao C., Chen Z. (2021). A review of current performance of rare earth metal-doped barium zirconate perovskite: The promising electrode and electrolyte material for the protonic ceramic fuel cells. Prog. Solid State Chem..

[2] Le L.Q., Hernandez C.H., Rodriguez M.H., Zhu L., Duan C., Ding H., O’Hayre R.P., Sullivan N.P. (2021). Proton-conducting ceramic fuel cells: Scale up and stack integration. J. Power Source.

[3] Zakowsky N., Williamson S., Irvine J.T.S. (2005). Elaboration of CO_2_ tolerance limits of BaCe_0.9_Y_0.1_O_3–δ_ electrolytes for fuel cells and other applications. Solid State Ion..

[4] Ma X., Dai J., Zhang H., Reisner D.E. (2005). Protonic conductivity nanostructured ceramic film with improved resistance to carbon dioxide at elevated temperatures. Surf. Coat. Technol..

[5] Radojković A., Žunić M., Savić S.M., Branković G., Branković Z. (2013). Enhanced stability in CO_2_ of Ta doped BaCe_0.9_Y_0.1_O_3−δ_ electrolyte for intermediate temperature SOFCs. Ceram. Int..

[6] Radojković A., Žunić M., Savić S.M., Perać S., Golić D.L., Branković Z., Branković G. (2019). Co-doping as a strategy for tailoring the electrolyte properties of BaCe_0.9_Y_0.1_O_3–δ_. Ceram. Int..

[7] Melnik J., Luo J.J., Chuang K.T., Sanger A.R. (2008). Stability and electric conductivity of barium cerate perovskites co-doped with praseodymium. Open Fuels Energy Sci. J..

[8] Dudek M., Lis B., Lach R., Daugėla S., Šalkus T., Kežionis A., Mosiałek M., Socha R.P., Morgiel J., Gajek M. (2019). Ba_0.95_Ca_0.05_Ce_0.9_Y_0.1_O_3_ as an electrolyte for proton-conducting ceramic fuel cells. Electrochim. Acta.

[9] Dudek M., Lis B., Lach R., Daugėla S., Šalkus T., Kežionis A., Mosiałek M., Sitarz M., Rapacz-Kmita A., Grzywacz P. (2020). Samples of Ba_1−x_Sr_x_Ce_0.9_Y_0.1_O_3−δ_, 0 < x < 0.1, with improved chemical stability in CO_2_-H_2_ gas-involving atmospheres as potential electrolytes for a proton ceramic fuel cell. Materials.

[10] Bello I.T., Zhai S., Zhao S., Li Z., Yu N., Ni M. (2021). Scientometric review of proton-conducting solid oxide fuel cells. Int. J. Hydrogen Energy.

[11] Ferguson K., Dubois A., Albrecht K., Braun R.J. (2021). High performance protonic ceramic fuel cell systems for distributed power generation. Energy Convers. Manag..

[12] Majhi S.M., Behura S.K., Bhattacharjee S., Singh B.P., Chongdar T.K., Gokhale N.M., Besra L. (2011). Anode supported solid oxide fuel cells (SOFC) by electrophoretic deposition. Int. J. Hydrogen Energy.

[13] Marrony M. (2016). Proton-Conducting Ceramics: From Fundamentals to Applied Research.

[14] Taillades G., Batocchi P., Essoumhi A., Taillades M., Jones D.J., Rozière J. (2011). Engineering of porosity, microstructure and electrical properties of Ni–BaCe_0.9_Y_0.1_O_2.95_ cermet fuel cell electrodes by gelled starch porogen processing. Microporous Mesoporous Mater..

[15] Agarwal V., Liu M. (1997). Electrochemical properties of BaCe0.8Gd0.2O3 electrolyte films deposited on Ni-BaCe_0.8_Gd_0.2_O_3_ substrates. J. Electrochem. Soc..

[16] Essoumhi A., Taillades G., Taillades-Jacquin M., Jones D.J., Rozière J. (2008). Synthesis and characterization of Ni-Cermet/Proton conducting thin film electrolyte symmetrical assemblies. Solid State Ion..

[17] Chevallier L., Zunic M., Esposito V., Di Bartolomeo E., Traversa E. (2009). A wet-chemical route for the preparation of Ni–BaCe_0.9_Y_0.1_O_3−δ_ Cermet anodes for IT-SOFCs. Solid State Ion..

[18] Zunic M., Chevallier L., Deganello F., D’Epifanio A., Licoccia S., Di Bartolomeo E., Traversa E. (2009). Electrophoretic deposition of Dense BaCe_0.9_Y_0.1_O_3−x_ electrolyte thick-films on Ni-based anodes for intermediate temperature solid oxide fuel cells. J. Power Source.

[19] Cao D., Zhou M., Yan X., Liu Z., Liu J. (2021). High performance low-temperature tubular protonic ceramic fuel cells based on barium cerate-zirconate electrolyte. Electrochem. Commun..

[20] Taillades G., Pers P., Mao V., Taillades M. (2016). High performance anode-supported proton ceramic fuel cell elaborated by wet powder spraying. Int. J. Hydrogen Energy.

[21] Anggia E., Shin E.-K., Nam J.-T., Park J.-S. (2020). Fabrication of ceramic composite anode at low temperature for high performance protonic ceramic fuel cells. Ceram. Int..

[22] Bae H., Choi G.M. (2015). Novel modification of anode microstructure for proton-conducting solid oxide fuel cells with BaZr_0.8_Y_0.2_O_3−δ_ Electrolytes. J. Power Source.

[23] Xiao J., Sun W., Zhu Z., Tao Z., Liu W. (2012). Fabrication and characterization of anode-supported dense BaZr_0.8_Y_0.2_O_3−δ_ Electrolyte membranes by a dip-coating process. Mater. Lett..

[24] Lis B., Dudek M., Kluczowski R., Krauz M., Kawalec M., Mosiałek M., Lach R. (2018). Physicochemical properties of ceramic tape involving Ca_0.05_ Ba_0.95_Ce_0.9_Y_0.1_O_3_ as an electrolyte designed for electrolyte-supported solid oxide fuel cells (IT-SOFCs). J. Therm. Anal. Calorim..

[25] Dudek M., Lis B., Kocyło E., Rapacz-Kmita A., Mosiałek M., Gajek M., Lach R., Presto S., Viviani M., Carpanese M.P. (2019). Utilisation of methylcellulose as a shaping agent in the fabrication of Ba_0.95_Ca_0.05_Ce_0.9_Y_0.1_O_3_ proton-conducting ceramic membranes via the gelcasting method. J. Therm. Anal. Calorim..

[26] James F., Roos M. (1975). MINUIT: A system for function minimization and analysis of the parameter errors and corrections. Comput. Phys. Commun..

[27] Mosiałek M., Nowak P., Dudek M., Mordarski G. (2014). Oxygen reduction at the Ag|Gd_0.2_Ce_0.8_O_1.9_ interface studied by electrochemical impedance spectroscopy and cyclic voltammetry at the silver point electrode. Electrochim. Acta.

[28] Kim H., Kim B., Lee J., Ahn K., Kim H.R., Yoon K.J., Kim B.K., Cho Y.W., Lee H.W., Lee J.H. (2014). Microstructural adjustment of Ni–BaCe_0.9_Y_0.1_O_3−δ_ cermet membrane for improved hydrogen permeation. Ceram. Int..

[29] Hao X., Han D., Wang J., Liu Y., Rooney D., Sun W., Qiao J., Wang Z., Sun K. (2015). Co-tape casting fabrication, field assistant sintering and evaluation of a coke resistant La_0.2_Sr_0.7_TiO_3_–Ni/YSZ functional gradient anode supported solid oxide fuel cell. Int. J. Hydrogen Energy.

[30] Myung J.H., Ho Shin T., Kim S.D., Park H.G., Moon J., Hyun S.H. (2015). Optimization of Ni–Zirconia based anode support for robust and high-performance 5 × 5 cm^2^ Sized SOFC via tape-casting/co-firing technique and nano-structured anode. Int. J. Hydrogen Energy.

[31] Agarkova E.A., Zadorozhnaya O.Y., Burmistrov I.N., Yalovenko D.V., Agarkov D.A., Rabotkin S.V., Solovyev A.A., Nepochatov Y.K., Levin M.N., Bredikhin S.I. (2021). Relationships between mechanical stability of the anode supports and electrochemical performance of intermediate-temperature SOFCs. Mater. Lett..

[32] Fabbri E., Pergolesi D., Traversa E. (2010). Materials challenges toward proton-conducting oxide fuel cells: A critical review. Chem. Soc. Rev..

[33] Rainwater B.H., Liu M., Liu M. (2012). A more efficient anode microstructure for SOFCs based on proton conductors. Int. J. Hydrogen Energy.

[34] Nasani N., Ramasamy D., Antunes I., Perez J. (2015). Electrochemical behaviour of Ni-BZO and Ni-BZY cermet anodes for Protonic Ceramic Fuel Cells (PCFCs)–A comparative study. Electrochim. Acta.

[35] Drożdż-Cieśla E., Wyrwa J., Broś J., Rękas M. (2012). Structural, microstructural, thermal, and electrical properties of Ni/YSZ Cermet materials. J. Therm. Anal. Calorim..

[36] Prakash B.S., Kumar S.S., Aruna S.T. (2014). Properties and development of Ni/YSZ as an anode material in solid oxide fuel cell: A review. Renew. Sust. Energy Rev..

[37] Marinšek M., Pejovnik S., Maček J. (2007). Modelling of electrical properties of Ni-YSZ composites. J. Eur. Ceram..

[38] Dailly J., Marrony M., Taillades G., Taillades-Jacquin M., Grimaud A., Mauvy F., Louradour E., Salmi J. (2014). Evaluation of proton conducting BCY10-based anode supported cells by co-pressing method: Up-scaling, performances and durability. J. Power Source.

[39] Baquero T., Escobar J., Frade J., Hotza D. (2013). Aqueous tape casting of micro and nano YSZ for SOFC electrolytes. Ceram. Int..

[40] Narendar N., Mather G.M., Dias P.A.N., Duncan P.F. (2013). The importance of phase purity in Ni–BaZr_0.85_Y_0.15_O_3−δ_ cermet anodes—Novel nitrate-free combustion route and electrochemical study. RSC Adv..

[41] Zunic M., Chevallier L., Radojkovic A., Brankovic G., Brankovic Z., Bartolomeo E.D. (2011). Influence of the ratio between Ni and BaCe_0.9_Y_0.1_O_3−δ_ on microstructural and electrical properties of proton conducting Ni–BaCe_0.9_Y_0.1_O_3−δ_ anodes. J. Alloys Compd..

[42] Tomczyk P., Zurek S., Mosiałek M. (2009). Effect of time and polarization on kinetics of the oxygen electrode reaction at an Au Ι YSZ interface. J. Electroceram..

[43] Drożdż-Cieśla E., Wrwa J., Pyda W., Rękas M. (2012). A new method of preparing Ni / YSZ cermet materials. J. Mater. Sci..

[44] Macdonald J.R. (1987). Impedance Spectroscopy.

[45] Mosiałek M., Dudek M., Michna A., Tatko M., Kędra A., Zimowska M. (2014). Composite cathode materials Ag-Ba_0_.5Sr0.5Co0.8Fe0.2O3 for solid oxide fuel cells. J. Solid State Electrochem..

[46] Kubota M., Okanishi T., Muroyama H., Matsui T., Eguchi K. (2015). Microstructural evolution of Ni–YSZ cermet anode under thermal cycles with redox treatments. J. Electrochem. Soc..

[47] Nasani N., Wang Z.J., Willinger M.G., Yaremchenko A.A., Fagg D.P. (2014). In-Situ redox cycling behaviour of Ni–BaZr_0.85_Y_0.15_O_3−δ_ cermet anodes for protonic ceramic fuel cells. Int. J. Hydrogen Energy.

[48] Shen C.T., Lee Y.H., Xie K., Yen C.P., Jhuang J.W., Lee K.R., Lee S.W., Tseng C.J. (2017). Correlation between microstructure and catalytic and mechanical properties during redox cycling for Ni-BCY and Ni-BCZY composites. Ceram. Int..

[49] Matsumoto H., Nomura I., Okada S., Ishihara T. (2008). Intermediate-temperature solid oxide fuel cells using perovskite-type oxide based on barium cerate. Solid State Ion..

[50] Rashid N.L.R.M., Samat A.A., Jais A.A., Somalu M.R., Muchtar A., Baharuddin N.A., Wan Isahak W.N.R. (2019). Review on zirconate-cerate-based electrolytes for proton-conducting solid oxide fuel cell. Ceram. Int..

